# The effects of sensory stimulation therapy in patients with sleep disorders: a scoping review

**DOI:** 10.3389/fnins.2025.1682267

**Published:** 2025-10-03

**Authors:** Zihan Qu, Xuefeng Sun, Xiaotu Zhang, Jiawei Yin, Xinye Zhang, Haifeng Zhao, Hongshi Zhang

**Affiliations:** ^1^School of Nursing, Changchun University of Chinese Medicine, Changchun, China; ^2^Department of Rehabilitation, The Second People’s Hospital of Dalian, Dalian, China

**Keywords:** sleep disorders, sensory stimulation, music therapy, light therapy, aromatherapy, massage therapy, scoping review

## Abstract

**Background:**

Sleep disorders are prevalent, affecting 27% of the population and linked to health issues like cognitive impairments and cardiovascular diseases. Current treatments have limitations, prompting interest in sensory stimulation therapy as an alternative approach.

**Objective:**

This scoping review aims to explore the effectiveness of sensory stimulation therapy as an intervention for improving sleep disorders, as well as its range of applicability, by synthesizing existing research.

**Methods:**

Using the methodological framework of Arksey and O’Malley and following the PRISMA guidelines, both keywords and free-text searches were conducted. Articles meeting the inclusion criteria were then selected from the China National Knowledge Infrastructure Database, Wanfang Knowledge Service Platform Database, PubMed, Embase, Web of Science and Cochrane Library databases. Each article was screened and analyzed according to specific research elements.

**Results:**

A total of 20 randomized controlled trials, involving 1489 participants across 2 types of sensory stimulation therapy, namely multi-sensory and single-sensory stimulations, were included in the study. The main sensory stimuli applied were auditory, visual, olfactory and tactile, while the intervention methods included music therapy, light therapy, aromatherapy and massage therapy. The intervention duration, frequency and cycles varied considerably, but most studies implementing individual sessions lasting 20–60 min, at least three sessions per week. Fourteen methods were used to assess sleep quality, and the majority of studies applied two or more assessment methods. Completion rates were also high, with 85%–100% of patients completing over 80% of the intervention protocol. Interestingly, 19 studies reported no adverse events. Overall, sensory stimulation therapy had a positive impact on sleep quality, particularly in aspects such as sleep latency, total sleep time, and subjective sleep experience.

**Conclusion:**

The results indicated that sensory stimulation therapy is a safe, feasible and beneficial intervention for improving sleep quality in patients with sleep disorders. However, several limitations were identified in the included studies. Future large-sample, multi-center, high-quality RCTs are needed to further verify the efficacy of this therapy, and provide stronger evidence for their clinical application.

**Systematic review registration:**

https://doi.org/10.17605/OSF.IO/KQ2XT

## 1 Background

Sleep disorders refer to abnormalities in sleep quality and quantity as a result of different factors, such as insufficient sleep, heightened awareness of sleep or abnormal movements during sleep, that impair daytime functioning (Pavlova and Latreille, 2019). The International Classification of Sleep Disorders (ICSD) classifies sleep disorders into seven main categories, namely insomnia, sleep-related movement disorders, sleep-related breathing disorders, circadian rhythm sleep-wake disorders, central sleep apnea, atypical sleep and other sleep-related disorders (Sateia, 2014). According to the World Health Organization, the prevalence of sleep disorders is as high as 27%, with an upward trend being observed every year (Brewster et al., 2022). Research indicates that poor sleep quality or insufficient sleep disrupts the body’s circadian rhythm, thereby increasing the risk of developing various health conditions, such as cognitive impairments and cardiovascular diseases (Redline et al., 2023). Additionally, with increasing age, the natural decline in the function of the endogenous circadian rhythm system often results in typical problems such as difficulty falling asleep, sleep fragmentation, and early morning awakening, due to decreased melatonin secretion and phase advance of the circadian rhythm. Consequently, sleep disorders are particularly prevalent in the elderly population (Taillard et al., 2021).

The main treatments for sleep disorders currently include pharmacological interventions and cognitive-behavioral therapy (Wilson et al., 2019). Specifically, medication options encompass benzodiazepines, sedatives and melatonin, amongst others, although their prolonged use may lead to adverse effects such as tolerance, dependence and addiction (Asarnow, 2020). In particular, extended use of certain drugs may also impair the cognitive functions of patients. On the other hand, cognitive behavioral therapy has shown significant therapeutic efficacy, but its long treatment duration, high demands on patient self-management and inconsistent outcomes have limited its widespread clinical application (Liu et al., 2024).

In this context, sensory stimulation therapy offers a promising alternative, especially due to its safety, affordability, broad applicability, flexibility and the potential to avoid the adverse effects associated with medications. Originating in the 1950s, sensory stimulation can be divided into single-sensory and multi-sensory stimulation, often involves various methods, such as light therapy, aromatherapy, music therapy and massage that use light effects, relaxing scents, soothing music and tactile stimuli to engage one or more of a patient’s senses (i.e., sight, smell, hearing, touch and taste) (Lombardi et al., 2002). In fact, it can effectively regulate central nervous system activity to improve patients’ negative behaviors and psychological symptoms. Therefore, it is widely used in the symptomatic management and clinical care of patients with Alzheimer’s disease, cognitive impairment, and other conditions. As far as sleep disorders are concerned, studies have further demonstrated that this therapy can enhance sleep structure and quality in patients, thereby improving their quality of life and yielding better therapeutic effects compared with conventional drug therapy (Wang and Cheng, 2024).

Despite the growing number of sensory stimulation interventions for sleep disorders, there is limited comprehensive analysis regarding their types, duration, evaluation methods and effectiveness. Additionally, systematic application summaries and practical guidelines are also lacking. Therefore, this study employs a scoping review methodology to systematically examine and analyze relevant studies from both domestic and international sources in order to provide useful insights and reference points for healthcare professionals engaged in clinical practice and related research.

## 2 Methods

This review used the methodological framework of Arksey and O’Malley (2005) which consists of the following five steps: (1) identifying the research question, (2) retrieving relevant studies, (3) selecting studies, (4) creating data graphs, (5) organizing, summarizing and reporting the results. It also follows the guidelines for the preferred reporting items for systematic reviews and meta-analyses (PRISMA) (Tricco et al., 2018). This review has been registered on the OSF platform under the registration number https://doi.org/10.17605/OSF.IO/KQ2XT.

### 2.1 Formulation of research questions

The research questions guiding this study were as follows: (1) What types of sensory stimulation therapy interventions are used for patients with sleep disorders, and what are their main components? (2) How should sensory stimulation therapy interventions for sleep disorders be scheduled in terms of timing, frequency and duration? (3) What methods are used to assess the sleep quality of patients undergoing sensory stimulation therapy and what are the outcomes of these interventions?

### 2.2 Inclusion and exclusion criteria for literature

The following inclusion criteria were applied: (1) The research participants had to meet the diagnostic criteria for sleep disorders (American Academy of Sleep Medicine, 2014). (2) Patients with sleep disorders aged 18 years or older. (3) The exposures or interventions, including combined ones, involved sensory stimulation therapy. (4) The study design was a randomized controlled trial (RCT).

Studies were excluded if: (1) The literature did not describe the content and efficacy of the sensory stimulation therapy intervention or lacked a detailed description of the intervention methods. (2) The literature was not published in Chinese or English. (3) The literature was not accessible in full. (4) They were reviews, guidelines, conference abstracts, symposium abstracts and research protocols.

### 2.3 Search the database

The databases China National Knowledge Infrastructure Database (CNKI), Wanfang Knowledge Service Platform Database (WFSD), PubMed, Embase, Web of Science and the Cochrane Library for randomized controlled clinical trials were searched using only human trials and RCTs as the search criteria. The search period extended from the databases’ inception to May 1, 2025.

### 2.4 Search strategies

A combination of subject terms and free terms was used, with the search scope set to title, abstract and keyword fields to ensure effective and comprehensive results. The search terms included “sleep disorders,” “insomnia,” “restless legs syndrome,” “sleep apnea hypopnea syndrome,” “aromatherapy,” “massage,” “music therapy,” “light therapy,” and “audio-visual.” The complete and detailed information of all search strategies is provided in the Supplementary Appendix A.

### 2.5 Literature screening and data extraction

All retrieved literature was imported into EndNote 20 software to remove duplicates. Two trained researchers then independently screened the titles and abstracts, after which they reviewed the full texts of literature that met the inclusion criteria before cross-checking each other’s findings. The following data were then jointly extracted from the included literature by both researchers: author, country and year of publication, study type, sample size, participant age, sensory modalities involved, intervention method, duration of intervention and outcome assessment methods. In cases of disagreement, a third researcher was consulted until a consensus was reached. Additionally, researchers referred to supplemental information provided in the included studies (e.g., clinical trial registration numbers) to obtain more detailed data.

### 2.6 Literature quality evaluation

The quality of the literature was independently assessed and cross-checked by two researchers. The evaluation, which was performed using the risk bias assessment tools from the Cochrane Systematic Review Manual (Cumpston et al., 2019), included the following six assessment criteria: (1) randomization methods; (2) concealment of allocation; (3) blinding of participants and intervention providers; (4) completeness of outcome data; (5) selective reporting; and (6) other potential biases. In this case, each criterion was rated as “low risk,” “high risk,” or “unclear.”

## 3 Results

### 3.1 Results of literature search

**FIGURE 1:**
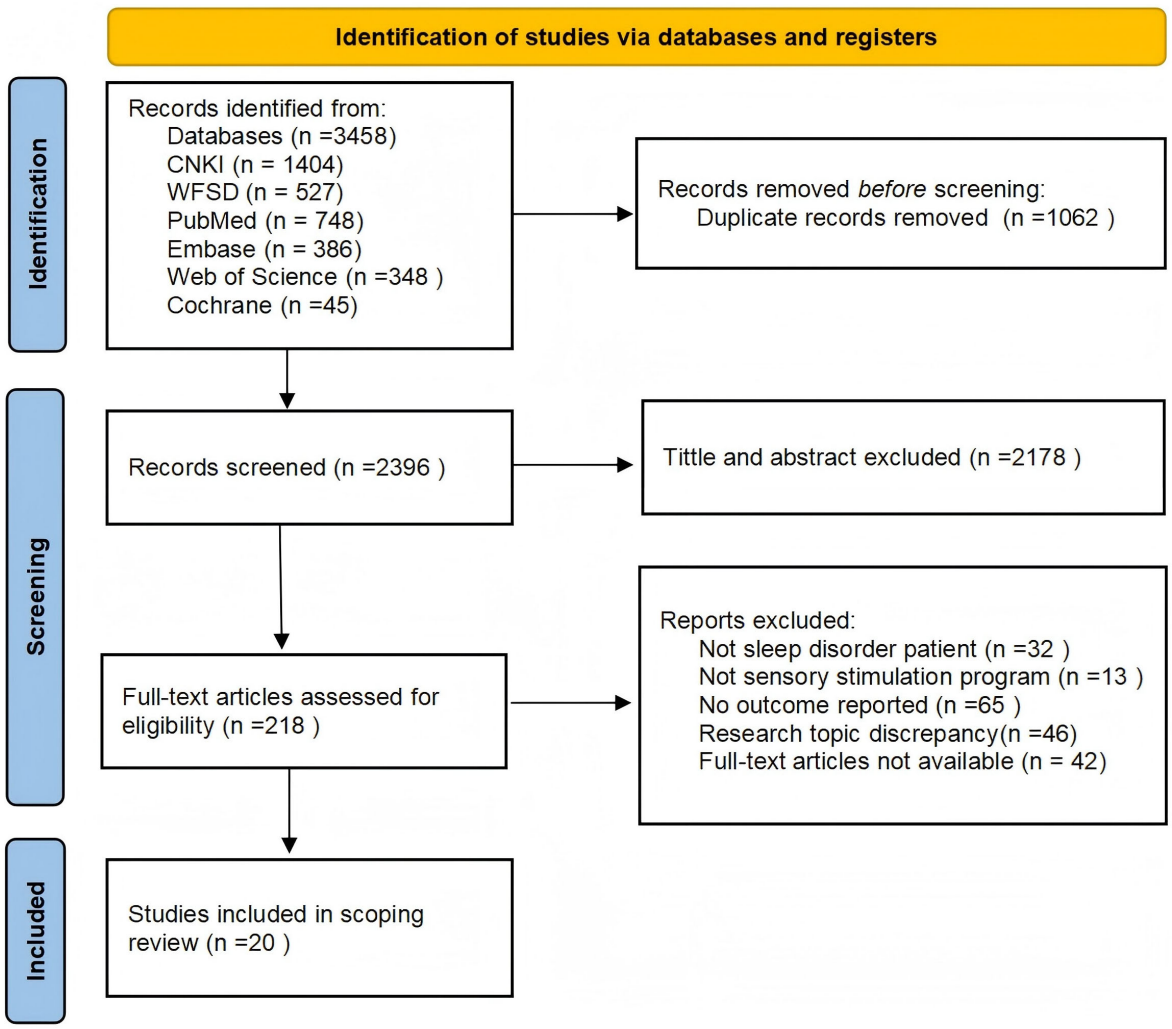
Preferred reporting items for systematic reviews and meta-analyses (PRISMA) flow diagram.

The initial search identified 3458 articles: 1404 from the CNKI database, 527 from the Wanfang Knowledge Service Platform database, 748 from PubMed, 386 from Embase, 348 from Web of Science and 45 from the Cochrane Library. From these, 1062 duplicate articles were removed, and after reviewing the titles, abstracts, and full texts of the remaining 2396 ones, review articles, guidelines, conference abstracts, articles on unrelated topics as well as those without full-text access were excluded. Eventually, a total of 20 articles were included (Ayik and Özden, 2018; Bean et al., 2022; Cheraghbeigi et al., 2019; Dos Reis Lucena et al., 2021; Genç et al., 2020; Ghasemi et al., 2021; Jespersen et al., 2019; Kawabata et al., 2020; Kim et al., 2022; Li et al., 2023; Lund et al., 2020; Oliveira et al., 2012; Oshvandi et al., 2021; Pattison et al., 2024; Rafii et al., 2020; Tang H. J. et al., 2021; Wang and Zhong, 2021; Wang Y. et al., 2024; Yang et al., 2024; Yoon et al., 2023). A flowchart illustrating the literature selection process is provided in Figure 1.

### 3.2 Inclusion criteria and quality assessment of the literature

Following a comprehensive review of the literature, 20 studies conducted across various countries were included: Iran (*n* = 4) (Cheraghbeigi et al., 2019; Ghasemi et al., 2021; Oshvandi et al., 2021; Rafii et al., 2020), China (*n* = 4) (Li et al., 2023; Wang and Zhong, 2021; Wang Y. et al., 2024; Yang et al., 2024), Turkey (*n* = 2) (Ayik and Özden, 2018; Genç et al., 2020), South Korea (*n* = 2) (Kim et al., 2022; Yoon et al., 2023), Brazil (*n* = 2) (Dos Reis Lucena et al., 2021; Oliveira et al., 2012), Denmark (*n* = 2) (Jespersen et al., 2019; Lund et al., 2020), the United States (*n* = 1) (Tang H. J. et al., 2021), Australia (*n* = 1) (Bean et al., 2022), the United Kingdom (*n* = 1) (Ghasemi et al., 2021) and Japan (*n* = 1) (Cheraghbeigi et al., 2019). All studies involved participants aged 18 years or older, with five focusing on middle-aged or elderly patients (Bean et al., 2022; Li et al., 2023; Oliveira et al., 2012; Tang H. J. et al., 2021; Yang et al., 2024). In addition, all studies involved randomized controlled trials, and most had sample sizes between 30 and 150 participants (*n* = 19) (Ayik and Özden, 2018; Bean et al., 2022; Cheraghbeigi et al., 2019; Dos Reis Lucena et al., 2021; Genç et al., 2020; Ghasemi et al., 2021; Jespersen et al., 2019; Kawabata et al., 2020; Kim et al., 2022; Li et al., 2023; Lund et al., 2020; Oliveira et al., 2012; Oshvandi et al., 2021; Pattison et al., 2024; Rafii et al., 2020; Tang H. J. et al., 2021; Wang and Zhong, 2021; Wang Y. et al., 2024; Yang et al., 2024), except for one in which less than 30 participants were included (Yoon et al., 2023). Table 1 presents the basic information of the included studies. Furthermore, of the included studies, five did not report allocation concealment, fourteen did not mention or apply blinding and two did not address the completeness of outcome data (Table 2). A visual representation of the risk of bias assessment is shown in Supplementary Appendix B.

**TABLE 1:** Characteristics of the included studies (*n* = 20).

References	Method	Country of origin	Sample size	Age	Types of sensory stimulation	Senses	Intervention	Measure	Completion rate
Bean et al., 2022	RCT	Australia	101	≥18	Multi-sensory stimulation	Visual, auditory	Audio therapy + light therapy: listen to specific relaxation audio tracks and wear light-emitting glasses for 20 min per day, for 6 weeks. Control group: audio therapy.	ABCD	100%
Tang H. J. et al., 2021	RCT	The United States	31	≥60	Multi-sensory stimulation	Visual, auditory	Auditory stimulation + light stimulation: low-frequency audio and dim light are administered for 30 min per day for 3 weeks. Control group: placebo.	ABE	96%
Wang Y. et al., 2024	RCT	China	81	43–50	Multi-sensory stimulation	Auditory, tactile	Massage + music therapy: head and facial massage, back shu point massage combined with the five element music therapy, 30 min per session, for 2 weeks. Control group: group 1 (massage), group 2 (music therapy).	EF	100%
Cheraghbeigi et al., 2019	RCT	Iran	150	18–65	Multi-sensory stimulation	Olfactory, tactile	Massage + aromatherapy: use lavender essential oil for hand and foot massage, 20 min per day, for 1 week. Control group: group 1 (massage), group 2 (routine care).	E	100%
Kawabata et al., 2020	RCT	Japan	74	≥18	Multi-sensory stimulation	Olfactory, tactile	Massage + aromatherapy: hand and foot massage using lavender essential oil, 30 min per day. Control group: routine care.	G	100%
Rafii et al., 2020	RCT	Iran	105	≥18	Multi-sensory stimulation	Olfactory, tactile	Massage + aromatherapy: use lavender essential oil for massage, 20 min per session, 3 times per week. Control group: group 1 (placebo), group 2 (routine care).	E	100%
Oshvandi et al., 2021	RCT	Iran	105	30–75	Multi-sensory stimulation	Olfactory, tactile	Massage + aromatherapy: use lavender essential oil for foot massage, 18 min per session, 3 times per week, for 3 weeks. Control group: massage.	EH	100%
Ayik and Özden, 2018	RCT	Turkey	96	≥18	Multi-sensory stimulation	Olfactory, tactile	Massage + aromatherapy: back massage using lavender essential oil, 10 min per session. Control group: routine care.	G	100%
Ghasemi et al., 2021	RCT	Iran	105	≥18	Multi-sensory stimulation	Olfactory, tactile	Massage + aromatherapy: use lavender essential oil for foot reflexology massage, 30 min per session, 3 times per week, for 8 weeks. Control group: group 1 (massage), group 2 (placebo).	H	100%
Pattison et al., 2024	RCT	The United Kingdom	34	≥18	Multi-sensory stimulation	Olfactory, tactile	Massage + aromatherapy: a 20-min hand, foot, and back massage using a blend of essential oils per day. Control group: routine care.	GI	94%
Li et al., 2023	RCT	China	86	18–80	Multi-sensory stimulation	Olfactory, auditory	Five element music therapy + aromatherapy: give patients 1 h of listening to five element music every day, and instruct them to perform compound essential oil aromatherapy before going to bed, close their eyes, and take deep breaths, for 2 weeks. Control group: routine care.	E	100%
Wang and Zhong, 2021	RCT	China	72	55–79	Multi-sensory stimulation	Visual, tactile, olfactory, auditory	Multi-sensory stimulation therapy: the room background color is selected in a blue tone that helps with sleep, with a green tone as an accent. The room light is controlled with blinds, and the patient is given the opportunity to listen to the five-element music and light incense. They are also given head, abdominal, and back massages. The session lasts 60 min per session, and is conducted three times per week for 4 weeks. Control group: routine care.	EI	87%
Yang et al., 2024	RCT	China	72	18–65	Single-sensory stimulation	Tactile	Massage: provide head, abdomen, and back massages once a day for 2 weeks. Control group: drug therapy.	EK	100%
Oliveira et al., 2012	RCT	Brazil	44	50–65	Single-sensory stimulation	Tactile	Massage: provide patients with acupuncture point massage in traditional Chinese medicine, 1 h per session, 3 times per week, for 16 weeks. Control group: routine care.	AF	100%
Yoon et al., 2023	RCT	South Korea	14	≥18	Single-sensory stimulation	Visual	Morning light therapy: using a small light box to simulate morning light, patients stare at the light box for continuous viewing for 25 min per day for 2 weeks before 9 am. Control group: routine care.	BCELMN	100%
Kim et al., 2022	RCT	South Korea	56	≥18	Single-sensory stimulation	Visual	Light therapy: intervention is carried out using a high-brightness LED light box, with a light intensity of 10,000 lux. The patient is instructed on the use and precautions. 30 min per day, for 4 weeks. Control group: placebo.	ACEN	100%
Jespersen et al., 2019	RCT	Denmark	57	18–65	Single-sensory stimulation	Auditory	Music therapy: patients should listen to music for at least 30 min in bed before going to sleep, 30 min per day, for 3 weeks. Control group: group 1 (audiobook), group 2 (routine care).	ACEF	100%
Lund et al., 2020	RCT	Denmark	112	18–65	Single-sensory stimulation	Auditory	Music therapy: patients should listen to music for at least 30 min in bed before going to sleep, receive regular treatment and care, 30 min per day, for 4 weeks. Control group: routine care.	CE	92%
Dos Reis Lucena et al., 2021	RCT	Brazil	35	48–65	Single-sensory stimulation	Olfactory	Aromatherapy: before bedtime, give the patient lavender essential oil and use a small bottle containing the oil to make circular motions around the neck level for 2 min. Gently inhale the scent. Repeat the process 10 min later. Make sure the scent of the essential oil can be smelled during sleep. Once per day, continue for 4 weeks. Control group: placebo.	AEF	97%
Genç et al., 2020	RCT	Turkey	59	≥65	Single-sensory stimulation	Olfactory	Aromatherapy: before bedtime, drop a few drops of lavender essential oil on a 2 cm × 2 cm cotton pad and place it on a stand about 15–20 cm away from the nose, so that the patient can inhale it continuously during sleep at night. 1 time/day, for 4 weeks. Control group: routine care.	E	100%

A, insomnia severity index (ISI); B, sleep diary; C, ActiGraph wGT3X-BT; D, patient-reported outcomes measurement system (PROMIS); E, Pittsburgh sleep quality index (PSQI); F, Polysomnography (PSG); G, Richards-Campbell Sleep Questionnaire (RCSQ); H, International Restless Legs Syndrome Rating Scale (IRLS); I, Bispectral Index Score (BIS); J, Athens Insomnia Scale (AIS); K, Hyperarousal scale (HAS); L, Morningness-Eveningness Questionnaire (MEQ); M, Stanford Sleepiness Scale (SSS); N, Epworth Sleepiness Scale (ESS).

**TABLE 2:** Quality assessment of the included literature.

References	Random sequence generation	Allocation concealment	Blinding of participants and personnel	Blinding of outcome assessment	Incomplete outcome data	Selective reporting	Other bias	Total no. high risk
Bean et al., 2022	Low	Low	High	Low	Low	Low	Low	1
Tang H. J. et al., 2021	Low	Unclear	Unclear	Low	Low	Low	Low	0
Wang Y. et al., 2024	Low	Low	High	Low	Low	Low	Low	1
Cheraghbeigi et al., 2019	Low	Low	High	Low	Low	Low	Low	1
Kawabata et al., 2020	Low	Low	High	Low	Unclear	Low	Low	1
Rafii et al., 2020	Low	Low	Low	Low	Low	Low	Low	0
Oshvandi et al., 2021	Low	Low	Low	Low	Low	Low	Low	0
Ayik and Özden, 2018	Low	Low	Low	Low	Low	Low	Low	0
Ghasemi et al., 2021	Low	Low	Low	Low	Low	Low	Low	0
Pattison et al., 2024	Low	Low	High	Low	Unclear	Low	Low	1
Li et al., 2023	Low	Unclear	High	Low	Low	Low	Low	1
Wang and Zhong, 2021	Low	Unclear	Unclear	Low	Low	Low	Low	0
Yang et al., 2024	Low	Low	Unclear	Low	Low	Low	Low	0
Oliveira et al., 2012	Low	Unclear	Unclear	Low	Low	Low	Low	0
Yoon et al., 2023	Low	Unclear	Unclear	Low	Low	Low	Low	0
Kim et al., 2022	Low	Low	Low	Low	Low	Low	Low	0
Jespersen et al., 2019	Low	Low	Unclear	Low	Low	Low	Low	0
Lund et al., 2020	Low	Low	High	Low	Low	Low	Low	1
Dos Reis Lucena et al., 2021	Low	Low	Low	Low	Low	Low	Low	0
Genç et al., 2020	Low	Low	High	Low	Low	Low	Low	1

### 3.3 Sensory stimulation therapy intervention plan for patients with sleep disorders

#### 3.3.1 Types and contents of sensory stimulation therapy intervention for patients with sleep disorders

Among the 20 included studies, 12 employed multi-sensory stimulation therapy (Ayik and Özden, 2018; Bean et al., 2022; Cheraghbeigi et al., 2019; Ghasemi et al., 2021; Kawabata et al., 2020; Li et al., 2023; Oshvandi et al., 2021; Pattison et al., 2024; Rafii et al., 2020; Tang H. J. et al., 2021; Wang and Zhong, 2021; Wang Y. et al., 2024), while 8 used single-sensory stimulation therapy (Dos Reis Lucena et al., 2021; Genç et al., 2020; Jespersen et al., 2019; Kim et al., 2022; Lund et al., 2020; Oliveira et al., 2012; Yang et al., 2024; Yoon et al., 2023), with both targeting auditory, visual, olfactory and tactile stimuli. The multi-sensory approach commonly combined two sensory modalities, such as auditory-visual, auditory-tactile, olfactory-tactile and olfactory-auditory combinations, with only one study using a combination of more than two sensory stimuli by incorporating visual, tactile olfactory and auditory components. Intervention methods included music therapy, light therapy, aromatherapy and massage. Specifically, the music therapy techniques commonly included the Five Elements Music Therapy and slow-paced music, although participants also had the option to choose their preferred music, which enhanced acceptance and compliance. In the case of light therapy, the options included morning and bright light therapy delivered through the use of light-emitting glasses, LED light boxes and adjustments to the indoor lighting. Finally, aromatherapy interventions frequently employed essential oils, such as lavender, orange and blended oils, while massage techniques usually included hand and foot massage, head massage, back massage and acupressure massage.

#### 3.3.2 Time and frequency of sensory stimulation therapy for patients with sleep disorders

All studies reported the duration of each intervention session (*n* = 20). In particular, among the 18 articles that detailed session length, the duration ranged from 20 to 60 min (Ayik and Özden, 2018; Bean et al., 2022; Cheraghbeigi et al., 2019; Ghasemi et al., 2021; Jespersen et al., 2019; Kawabata et al., 2020; Kim et al., 2022; Li et al., 2023; Lund et al., 2020; Oliveira et al., 2012; Oshvandi et al., 2021; Pattison et al., 2024; Rafii et al., 2020; Tang H. J. et al., 2021; Wang and Zhong, 2021; Wang Y. et al., 2024; Yang et al., 2024; Yoon et al., 2023), with most specifying a 20-min duration. In addition, two studies extended their aromatherapy sessions to span the entire night’s sleep (Dos Reis Lucena et al., 2021; Genç et al., 2020), thus requiring participants to inhale continuously throughout their nighttime sleep. Regarding intervention frequency, all participants received at least three sessions per week (*n* = 20). However, the intervention cycle varied considerably across studies. Specifically, of the 16 studies that specified the intervention cycle, one study reported a 16-weeks cycle (*n* = 1) (Oliveira et al., 2012), another one an 8-weeks cycle (Ghasemi et al., 2021) and a third one a 6-weeks cycle (*n* = 1) (Bean et al., 2022). The remaining studies indicated cycles of 4 weeks (*n* = 5) (Dos Reis Lucena et al., 2021; Genç et al., 2020; Kim et al., 2022; Lund et al., 2020; Wang and Zhong, 2021), 3 weeks (*n* = 3) (Jespersen et al., 2019; Oshvandi et al., 2021; Tang H. J. et al., 2021), 2 weeks (*n* = 4) (Li et al., 2023; Wang Y. et al., 2024; Yang et al., 2024; Yoon et al., 2023) and 1 week (*n* = 1) (Cheraghbeigi et al., 2019).

### 3.4 Assessment of sleep quality in patients receiving sensory stimulation therapy

In the 20 included studies, 14 different assessment methods were used to evaluate sleep quality in patients with sleep disorders. Of these, seven studies used only one assessment method (Ayik and Özden, 2018; Cheraghbeigi et al., 2019; Genç et al., 2020; Ghasemi et al., 2021; Li et al., 2023; Rafii et al., 2020), with the rest employing two or more methods to assess sleep quality (*n* = 14) (Bean et al., 2022; Dos Reis Lucena et al., 2021; Jespersen et al., 2019; Kim et al., 2022; Lund et al., 2020; Oliveira et al., 2012; Oshvandi et al., 2021; Pattison et al., 2024; Tang H. J. et al., 2021; Wang and Zhong, 2021; Wang Y. et al., 2024; Yang et al., 2024; Yoon et al., 2023). Furthermore, subjective evaluation indicators included PSQI, ISI and sleep diaries, while objective ones included PSG and the ActiGraph wGT3X-BT activity monitor. PSQI is a widely used subjective measure in clinical practice for assessing sleep disorders, and it specifically evaluates components such as sleep quality, sleep latency, sleep duration, normal sleep efficiency, sleep disorders, use of sleep medication and daytime function impairment (Chen et al., 2018). In contrast, ISI assesses the nature, severity and impact of insomnia over the past 2 weeks (Yu, 2010). Additionally, a sleep diary records sleep onset and wake times, the frequency of awakenings, self-assessed sleep quality and daytime alertness, thus providing a comprehensive reflection of a patient’s sleep quality (Dietch and Taylor, 2021). PSG, recognized as the gold standard for diagnosing sleep disorders (Zhang et al., 2019), uses multi-channel monitoring to continuously record bioelectrical and physiological changes during sleep. In particular, it captures parameters, such as EEG, EMG, ECG, EOG, SpO2 and respiration to objectively assess a patient’s sleep quality (Schyvens et al., 2024). Finally, the ActiGraph wGT3X-BT is a wearable device that records body movement, allowing long-term objective data to be collected in an economical and efficient way. In 2007, the American Academy of Sleep Medicine (AASM) recognized it as an effective and reliable assessment tool for evaluating sleep and circadian rhythm disorders (Sadeh and Acebo, 2002).

### 3.5 Effects of sensory stimulation therapy on patients with sleep disorders

This study investigated the feasibility and effectiveness of sensory stimulation therapy in improving sleep quality for patients with sleep disorders. (1) Feasibility: Sensory stimulation therapy demonstrated good safety and feasibility for patients with sleep disorders, and for most of them, it was practical and safe to conduct at least three sessions per week during hospitalization. In fact, out of 19 studies, none reported adverse events, with the only exception being one instance of mild photophobia after bright light therapy although no other serious adverse events were noted (Yoon et al., 2023). Furthermore, the researchers set an adherence criterion of completing more than 80% of the intervention plan, with the results indicating that 85%–100% of the patients met this criterion, thus demonstrating good compliance. In this case, the main barriers to treatment completion included early discharge, employment changes, relocation, children’s educational needs, lack of energy, aversion to essential oil scents and death. (2) Sleep quality: Sensory stimulation therapy significantly improved the sleep quality of patients with sleep disorders to different extents. Common evaluation indicators included sleep latency, total sleep time, sleep structure, sleep efficiency and subjective sleep experience. Of the 20 included studies, 15 reported notable improvements in sleep latency, total sleep time and subjective sleep experience following the intervention (*p* < 0.05) (Table 3).

**TABLE 3:** Comparison of sleep quality assessment between groups.

References	Sleep quality evaluation		
	Control	Intervention	*P*-value
Bean et al., 2022	ISI: −6.73	ISI: −3.67	ISI: *p* = 0.009
Tang H. J. et al., 2021	ISI: 13.9 ± 4.3 PSQI: 8.9 ± 3.3	ISI: 11.5 ± 5.4 PSQI: 8.3 ± 4.3	ISI: *p* = 0.354 PSQI: *p* = 0.860
Wang Y. et al., 2024	Group 1: PSQI: 14.89 ± 1.15 PSG: sleep latency (min): 28.13 ± 6.84 Number of awakenings (times): 3.91 ± 0.88 REM (min): 15.62 ± 4.01 Total sleep time (min): 332.00 ± 21.97 Awakening/sleep ratio (%): 6.96 ± 5.07 Sleep efficiency (%): 85.99 ± 6.13 Group 2: PSQI: 15.05 ± 1.83 PSG: sleep latency (min): 29.47 ± 7.15 Number of awakenings (times): 4.22 ± 1.21 REM (min): 14.86 ± 4.34 Total sleep time (min): 329.16 ± 23.88 Awakening/sleep ratio (%): 5.13 ± 4.12 Sleep efficiency (%): 85.58 ± 6.95	PSQI: 11.19 ± 1.06 PSG: sleep latency (min): 24.79 ± 5.01 Number of awakenings (times): 2.14 ± 0.49 REM (min): 18.53 ± 3.58 Total sleep time (min): 351.00 ± 19.74 Awakening/sleep ratio (%): 3.35 ± 3.01 Sleep efficiency (%): 88.65 ± 6.28	PSQI: *p* < 0.05 PSG: *p* < 0.05
Cheraghbeigi et al., 2019	Group 1: PSQI: 3 ± 1 Group 2: PSQI: 8 ± 2	PSQI: 3 ± 2	PSQI: *p* < 0.01 *P* = 0.59
Kawabata et al., 2020	RCSQ: 405.8 ± 102.8	RCSQ: 384.1 ± 92.8	RCSQ: *p* = 0.60
Rafii et al., 2020	Group 1: PSQI: 10.03 ± 2.44 Group 2: PSQI: 10.28 ± 3.01	PSQI: 8.45 ± 3.24	PSQI: *p* < 0.01
Oshvandi et al., 2021	PSQI: Subjective sleep quality: 1.62 ± 0.91 Sleep latency: 2.60 ± 0.84 Sleep duration: 2.77 ± 0.54 Sleep efficiency: 2.82 ± 0.45 Sleep disturbance: 1.45 ± 0.50 Use of sleep medication: 0.40 ± 0.84 Daytime dysfunction: 0.60 ± 0.65 IRLS: 21.22 ± 6.50	PSQI: Subjective sleep quality: 0.74 ± 0.44 Sleep latency: 1.42 ± 0.69 Sleep duration: 2.65 ± 0.63 Sleep efficiency: 2.40 ± 1.06 Sleep disturbance: 0.88 ± 0.32 Use of sleep medication: 0.02 ± 0.16 Daytime dysfunction: 0.28 ± 0.45 IRLS: 3.58 ± 2.80	PSQI: *p* < 0.001 IRLS: *p* < 0.001
Ayik and Özden, 2018	RCSQ: 42.80 ± 19.45	RCSQ: 66.82 ± 17.98	RCSQ: *p* = 0.000
Ghasemi et al., 2021	Group 1: IRLS: 16.80 ± 5.357 Group 2: IRLS: 19.51 ± 2.904	IRLS: 13.20 ± 4.880	IRLS: *p* < 0.05
Pattison et al., 2024	RCSQ: 47.6 ± 37.9	RCSQ: 50.4 ± 13.8	RCSQ: *p* < 0.05
Li et al., 2023	PSQI: Sleep quality: 1.54 ± 0.34 Time to fall asleep: 1.21 ± 0.20 Sleep duration: 1.24 ± 0.38 Sleep efficiency: 1.34 ± 0.14 Sleep disorders: 1.02 ± 0.31 Daytime functional impairment: 1.18 ± 0.22	PSQI: Sleep quality: 1.22 ± 0.21 Time to fall asleep: 1.51 ± 0.46 Sleep duration: 0.63 ± 0.81 Sleep efficiency: 0.84 ± 0.32 Sleep disorders: 1.12 ± 0.11 Daytime function impairment: 0.83 ± 0.18	PSQI: *p* < 0.05
Wang and Zhong, 2021	PSQI: 17.81 ± 2.07 AIS: 15.89 ± 3.18	PSQI: 6.82 ± 1.33 AIS: 11.64 ± 3.58	PSQI: *p* < 0.05 AIS: *p* < 0.05
Yang et al., 2024	PSQI: 7.45 ± 3.38 HAS: 6.23 ± 2.03	PSQI: 7.75 ± 3.46 HAS: 30.60 ± 9.72	PSQI: – HAS: *p* = 0.49
Oliveira et al., 2012	ISI: 14	ISI: 6.5	ISI: *p* = 0.001
Yoon et al., 2023	ESS: 6.7 ± 4.3 ISI: 11.3 ± 4.4 PSQI: 10.6 ± 2.9	ESS: 5.3 ± 3.4 ISI: 7.7 ± 2.0 PSQI: 9.6 ± 2.8	ESS: *p* = 0.025 ISI: *p* = 0.024 PSQI: *p* = 0.004
Kim et al., 2022	Actiwatch: Sleep latency (minutes): 62.28 (58.16) Total sleep time (minutes): 440.45 (121.56) PSQI: 11.72 (2.36) ISI: 12.69 (6.22) ESS: 3.17 (3.51)	Actiwatch: Sleep latency (minutes): 38.92 (28.19) Total sleep time (minutes): 456.74 (159.01) PSQI: 10.11 (2.10) ISI: 11.52 (7.11) ESS: 2.74 (2.82)	Actiwatch: *p* < 0.05 PSQI: *p* < 0.05 ISI: *p* < 0.05 ESS: *p* < 0.05
Jespersen et al., 2019	Group 1: ISI: 17.7 (3.3) PSQI: 10.4 (3.1) Group 1: ISI: 16.5 (4.9) PSQI: 11.2 (3.0)	ISI: 13.9 (5.3) PSQI: 8.7 (3.8)	ISI: *p* < 0.05 PSQI: *p* < 0.05
Lund et al., 2020	PSQI: 13.9	PSQI: 11.6	PSQI: *p* < 0.001
Dos Reis Lucena et al., 2021	ISI: 13.2 ± 4.8 PSQI: 9.4 ± 2.8 PSG: REML (min): 102.2 ± 65.8 Sleep efficiency: 75.6 ± 8.7 REM (%): 21.8 ± 4.6 WASO (min): 69.6 ± 34.3	ISI: 10.0 ± 4.4 PSQI: 7.5 ± 2.7 PSG: REML (min): 78.4 ± 26.5 Sleep efficiency: 82.1 ± 8.2 REM (%): 24.7 ± 4.5 WASO (min): 44.0 ± 28.2	ISI: *p* = 0.25 PSQI: *p* = 0.22 PSG: *p* < 0.05
Genç et al., 2020	PSQI: 8.00 ± 2.96	PSQI: 5.06 ± 2.51	PSQI: *p* < 0.001

ISI, insomnia severity index; PSQI, Pittsburgh sleep quality index; PSG, Polysomnography; RCSQ, Richards-Campbell Sleep Questionnaire; IRLS, International Restless Legs Syndrome Rating Scale; AIS, Athens Insomnia Scale; HAS, Hyperarousal scale; ESS, Epworth Sleepiness Scale.

## 4 Discussion

### 4.1 Interventions of sensory stimulation therapy

#### 4.1.1 Auditory stimulation

Soothing music is converted into neural signals via the cochlea, transmitted through the thalamus to the auditory cortex, and activates the amygdala, hippocampus, and other components of the limbic system. This process activates the parasympathetic nervous system, thereby reducing sympathetic nervous system arousal and inducing a state of relaxation (Šimić et al., 2021). In addition, auditory stimulation can also synchronize cortical activity through non-specific pathways, thereby enhancing slow-wave sleep (Bellesi et al., 2014). It is worth noting that the sleep-promoting effects of rhythmic auditory stimulation are based on a broad neurophysiological foundation. The mechanisms may involve regulating the balance of the autonomic nervous system through regular sound patterns, enhancing parasympathetic activity, and synchronizing brain electrical activity with a state of relaxation and sleep readiness (Wang et al., 2025). These mechanisms are to some extent universal across ages, which can be observed from the good response of individuals to rhythmic auditory signals in early developmental stages (Gou et al., 2025). Therefore, the application of rhythmic auditory stimulation interventions, such as music and white noise, to adult sleep disorders in clinical practice has its inherent physiological rationale. Studies have shown that music therapy, through targeted auditory stimulation, can modulate the key neurotransmitter systems involved in sleep regulation. Specifically, it promotes the release of dopamine, which is associated with pleasure and rewards and can indirectly improve sleep by alleviating psychological distress and enhancing emotional states (Wang X. et al., 2024). Additionally, music therapy can help relieve pain, stabilize emotions, and promote sleep by enhancing the secretion of endorphins and serotonin, the latter of which also serves as a precursor to melatonin. Research has also shown that music therapy can increase melatonin levels, a key hormone that directly regulates circadian rhythms and promotes sleep onset and maintenance (Dan et al., 2025; Le et al., 2025). By synergistically influencing these neurochemical pathways, music therapy can effectively reduce anxiety and depression, thereby creating a favorable neurobiological environment for improving sleep quality. For effective music therapy, slow-paced, soothing melodies at moderate volumes are recommended, while intense or fast-paced music is avoided as it may disrupt sleep.

#### 4.1.2 Visual stimulation

Light therapy is another cost-effective and easily manageable approach, and its effects involve two key pathways. The first is the image-forming visual pathway, where photic signals are received by the rod cells responsible for scotopic vision and the cone cells responsible for photopic and color vision in the retina, which are primarily involved in visual perception (Shi and Li, 2020). The second is the non-image-forming pathway, which is crucial for the regulation of the sleep-wake cycle. This pathway is mediated by the intrinsically photosensitive retinal ganglion cells (ipRGCs) in the retina, which express melanopsin and are particularly sensitive to short-wavelength blue light. The ipRGCs receive and integrate signals from the rods and cones, and they can also directly sense light. Their axons project directly to the suprachiasmatic nucleus (SCN) of the hypothalamus, which serves as the central circadian pacemaker in humans. Through this pathway, photic signals inhibit the SCN’s daytime suppression of melatonin secretion from the pineal gland, thereby effectively regulating the circadian rhythm, correcting sleep phase shifts, and improving daytime alertness and nighttime sleep quality (Blume et al., 2019). This is particularly beneficial for elderly patients who experience decreased endogenous melatonin secretion and reduced circadian rhythm amplitude due to aging, and serves as an effective non-pharmacological means to correct their advanced sleep-wake phase (such as early morning awakening). However, patients with eye conditions, such as light sensitivity or retinal degeneration, should avoid light therapy to prevent aggravating symptoms. In fact, in one of the included studies, a participant experienced dizziness and withdrew from the light therapy (Yoon et al., 2023).

#### 4.1.3 Tactile stimulation

Massage activates various tactile receptors, such as mechanoreceptors, through mechanical pressure on the skin and deep tissues. The resulting signals are transmitted to the somatosensory cortex in the brain via ascending pathways like the spinothalamic tract (Zhang and Wang, 2018). In addition, this stimulation can induce relaxation by promoting parasympathetic nervous system activity and reducing heart rate, blood pressure, and cortisol levels through spinal reflexes and effects on the autonomic nervous system (Diego and Field, 2009). In particular, when specific acupoints are stimulated, such as the Baihui and Taiyang points, it can help calm the mind and induce sleep.

#### 4.1.4 Olfactory stimulation

On the other hand, aromatherapy uses natural plant essential oils to stimulate emotion-related regions of the brain through the olfactory route, thereby enhancing the release of neurotransmitters. Unlike other sensory systems, a unique pathway for olfactory signals bypasses the thalamus and directly projects to the limbic system of the brain, particularly the amygdala and hippocampus, which are closely associated with emotions and memory (Sullivan et al., 2015). This direct neural connection enables odors to rapidly and intensely influence emotional states and psychophysiological arousal levels. Furthermore, certain components, such as volatile organic compounds, in essential oils can relax muscles, improve blood circulation and induce other physiological effects in patients, thus helping to reduce fatigue and enhance sleep quality (Tang Y. et al., 2021). Despite being effective, aromatherapy should nevertheless be used with caution for certain groups, such as pregnant women, children and individuals who may be sensitive to certain components of the essential oils (Nakajima et al., 2017).

Taken together, patients with sleep disorders often experience associated physical, cognitive and emotional challenges, including anxiety and depression. As such, special requirements and higher skills may be needed from the operator for selecting and implementing sensory stimulation therapy interventions. However, a systematic and standardized training model for sensory stimulation therapy is not yet present in clinical practice. Hence, developing structured training and assessment criteria for sensory stimulation therapy would support systematic learning by healthcare professionals and maximize the intervention’s effectiveness.

### 4.2 Types of sensory stimulation therapy

The results of this study showed that 8 articles applied single-sensory stimulation therapy (Dos Reis Lucena et al., 2021; Genç et al., 2020; Jespersen et al., 2019; Lund et al., 2020; Oliveira et al., 2012; Yang et al., 2024; Yoon et al., 2023), while 12 used multi-sensory stimulation therapy which integrated two or more sensory modalities (Ayik and Özden, 2018; Bean et al., 2022; Cheraghbeigi et al., 2019; Ghasemi et al., 2021; Kawabata et al., 2020; Li et al., 2023; Oshvandi et al., 2021; Pattison et al., 2024; Rafii et al., 2020; Tang H. J. et al., 2021; Wang and Zhong, 2021; Wang Y. et al., 2024). Interestingly, only one study combined multiple interventions specifically for sleep disorders (Wang and Zhong, 2021). Given the complexity and varied causes of sleep disorders, single-sensory therapies providing stimuli through a single sensory channel may have limited effects due to the isolated nature of the stimulus and variability among patients. In this context, research by Wang et al. (2024b), (Cheraghbeigi et al., 2019; Ghasemi et al., 2021; Oshvandi et al., 2021; Rafii et al., 2020) suggested that multi-sensory stimulations had a stronger impact than single-sensory ones as they more effectively alleviated sleep disorder symptoms while enhancing overall sleep quality and daytime function. For elderly patients with sleep disorders in particular, their sleep issues often coexist with multiple problems such as anxiety and cognitive decline (Xu et al., 2021). This multi-target and holistic intervention strategy may be more suitable than single-sensory interventions for addressing the complex pathophysiological conditions of this population, including their unique circadian rhythm disorders. Clinical settings have also been gradually introducing “sleep rooms” with the aim of integrating multiple sensory therapies in a single space to provide patients with combined treatments, thereby maximizing therapeutic efficacy while optimizing the time of patients and medical professionals. However, studies on this approach remains limited, with a need for evidence from large sample sizes and long-term follow-ups. Therefore, further research and clinical trials are needed to validate the “sleep room” approach for treating sleep disorders, with regular follow-ups also essential to confirm its long-time benefits.

### 4.3 Duration and frequency of sensory stimulation therapy interventions

Currently, there are considerable variations in the duration and frequency of sensory stimulation therapy used to treat sleep disorders. While all studies required a minimum of three complete interventions per week, the length of each training session varied between 20 and 60 min, possibly due to differences in intervention methods. Similarly, there was a wide range of intervention cycles, with most studies implementing cycles of 2–4 weeks, although some extended over several months. Given the diversity of patients in terms of physical conditions, recovery rates, and underlying causes of sleep disorders, establishing a standardized duration and frequency for interventions may help enhance the safety and efficacy of treatments. However, such standardization needs to be flexibly adjusted according to individual differences to ensure that patients do not receive excessive stimulation or insufficient intervention that could affect the treatment outcomes. Additionally, standardized, individualized intervention criteria would enable healthcare professionals to assess treatment effectiveness more accurately for developing precise treatment plans. Future research should therefore aim to define standard intervention durations and cycles for sensory stimulation therapy.

### 4.4 Assessment methods for sensory stimulation therapy

This study’s primary outcome measure was sleep quality, assessed across 20 studies using 14 different methods. The results clearly highlighted a lack of standardized tools for evaluating the impact of sensory stimulation therapy in patients with sleep disorders. The evaluation methods consisted of both subjective and objective measures, but only 8 of the included studies used a combination of both types of tools (Bean et al., 2022; Dos Reis Lucena et al., 2021; Jespersen et al., 2019; Lund et al., 2020; Oliveira et al., 2012; Wang and Zhong, 2021; Wang Y. et al., 2024; Yoon et al., 2023), with the remaining 12 relying solely on subjective ones (Ayik and Özden, 2018; Cheraghbeigi et al., 2019; Genç et al., 2020; Ghasemi et al., 2021; Kawabata et al., 2020; Kim et al., 2022; Li et al., 2023; Oshvandi et al., 2021; Pattison et al., 2024; Rafii et al., 2020; Tang H. J. et al., 2021; Yang et al., 2024). Among the subjective tools, the PSQI is arguably the most widely used internationally for assessing sleep quality during both daytime and night time in healthy individuals as well as those with sleep or mental health-related sleep issues (Chen et al., 2021). ISI and AIS are also commonly used to gauge the severity of sleep disorders, with the ISI and the AIS focusing on the past 2 weeks (Dietch and Taylor, 2021) and the past month, respectively (Chung et al., 2011). Finally, subjective measures, such as the SSS and ESS, assess a patient’s level of drowsiness, but while SSS assesses a patient’s current sleepiness level (Chiu et al., 2017), ESS measures the patient’s general daytime sleepiness (Gonçalves et al., 2023). Relying solely on subjective assessments is prone to bias due to individual perception, emotions, and past experiences. If assessors lack standardized training, this subjectivity may be further amplified, leading to a reduction in the consistency of the assessment results. This study found that 13 articles used two or more assessment methods to assess sleep quality (Bean et al., 2022; Dos Reis Lucena et al., 2021; Jespersen et al., 2019; Kim et al., 2022; Lund et al., 2020; Oliveira et al., 2012; Oshvandi et al., 2021; Pattison et al., 2024; Tang H. J. et al., 2021; Wang and Zhong, 2021; Wang Y. et al., 2024; Yang et al., 2024; Yoon et al., 2023), while 7 relied on a single tool (Ayik and Özden, 2018; Cheraghbeigi et al., 2019; Genç et al., 2020; Ghasemi et al., 2021; Kawabata et al., 2020; Li et al., 2023; Rafii et al., 2020). Despite the common use of multiple evaluation methods, significant heterogeneity remains in their scope and emphasis. Selecting sleep quality assessment tools should therefore be individualized and based on each patient’s specific symptoms and needs. Furthermore, to enhance the objectivity, accuracy and reliability of sleep quality assessment tools, large-scale studies are needed to develop and standardize outcome evaluation tools that align with clinical requirements.

### 4.5 Efficacy and safety of sensory stimulation therapy

The results of this study show that sensory stimulation therapy significantly improves sleep quality in patients with sleep disorders while being highly safe. As a non-invasive intervention, sensory stimulation therapy circumvents the side effects and risks of dependency associated with medication, while allowing patients to receive treatment in a relatively safe environment. This characteristic makes it particularly suitable for the elderly population, who have decreased physical function and metabolic capacity and are more sensitive to adverse drug reactions, providing a highly promising option for managing their age-related sleep and circadian rhythm disorders. Of the 20 studies that were included, most reported no adverse events, with only one noting a minor adverse effect related to the intervention (Yoon et al., 2023). Additionally, the equipment and materials required for sensory stimulation therapy are relatively simple, and the procedures are straightforward, thus making this approach highly feasible for clinical applications. The results further suggested that patients who received sensory stimulation therapy demonstrated high adherence to the treatment, with an overall high completion rate for the intervention. Common barriers during the intervention period included early discharge, job changes, relocation, children’s schooling needs, lack of energy, rejection of essential oil aroma and death. However, it should be noted that, except for aversion to the scent of essential oil, none of the barriers were directly related to the sensory stimulation therapy itself.

### 4.6 Quality assessment of studies

Most studies included in this review did not use or report blinding methods, which may introduce biases in publication and implementation. Blinding is critical for minimizing subjective bias and improving the reliability of clinical trial results, and hence, a lack of blinding undoubtedly weakens the credibility of research findings (Burkhardt et al., 2010). Sensory stimulation therapy poses challenges for effective concealment due to its distinctive characteristics. Future research should therefore seek to develop innovative blinding methods and techniques to enhance the objectivity and reliability of outcomes. For instance, in music therapy, sound processing technology could combine different melodies to create a “composite music” that is difficult to differentiate. Alternatively, a “white noise” lacking rhythm and melody could be used as a placebo.

## 5 Limitations

This study had several limitations: Firstly, the included participants were all adults, although adolescents may also frequently experience sleep disorders. For instance, Rezaei et al. (2023) conducted an intervention study on infants with sleep disorders using tactile stimulation, and the results showed that the latter could increase vagal activity, lower heart rate and cortisol levels, reduce the number of nighttime awakenings, extend continuous sleep and improve overall sleep quality. While some studies have confirmed that sensory stimulation can enhance sleep quality in adolescents with sleep disorders, research on the application of the therapy for this particular population remains limited. Future studies should consider the unique characteristics and needs of adolescents with sleep disorders in order to develop suitable sensory stimulation therapy intervention programs that are tailored to them. Secondly, the limited number and quality of clinical studies on the use of sensory stimulation therapy for sleep disorders, coupled with inconsistencies in intervention protocols and assessment tools, present challenges. Indeed, of the 20 included studies, variations in their intervention durations and frequencies precluded a comparison of their respective effects on sleep quality, leading to heterogeneous results. Future research could consider including large-sample randomized controlled trials to develop comprehensive sensory stimulation intervention programs that use both subjective and objective evaluation tools to assess effectiveness. Thirdly, the studies included in this review cover a diverse range of cultural and geographical backgrounds from multiple regions, including the Middle East, Europe, China, Australia, and others. While this enhances the generalizability of the results, it also implies the potential for cultural and lifestyle differences (such as diet and daily routines) that may shape distinct sleep patterns and influence patients’ preferences and acceptance of specific interventions. Future research should delve deeper into cultural adaptation adjustments, such as developing music libraries or aromatherapy formulations that are in line with local cultural contexts, to further improve the acceptability and effectiveness of the interventions.

## 6 Conclusion

Sensory stimulation therapy is a safe, feasible and beneficial intervention for improving sleep quality in patients with sleep disorders. However, research on its application is still in its exploratory stages, and standardized, scientifically-validated intervention protocols have yet to be established. Additionally, there is a need for evaluation frameworks that combine subjective and objective measures. Future research should focus on developing structured and evidence-based intervention strategies, establishing uniform evaluation criteria and conducting large-sample, multi-center RCTs to assess the efficacy of sensory stimulation therapy for patients with sleep disorders.

## Data Availability

The original contributions presented in this study are included in this article/Supplementary material, further inquiries can be directed to the corresponding author.
