# Supplementary material for: The effects of sensory stimulation therapy in patients with sleep disorders: a scoping review
Source: Front Neurosci. 2025 Oct 3;19:1682267. doi: 10.3389/fnins.2025.1682267 (PMC12533477; doi:10.3389/fnins.2025.1682267)
Supplement: Supplementary file 1 [file Data_Sheet_1.docx]

# **Supplementary Appendix A: search strategy**

Pubmed

| #1 | Sleep Disorders OR Insomnia OR Restless Legs Syndrome OR Sleep Apnea, Obstructive OR Disorders, Sleep Wake OR Sleep Wake Disorder OR Wake Disorders, Sleep OR Disorders, Sleep OR Sleep Disorder OR Long Sleeper Syndromes OR Sleeper Syndromes, Long OR Syndromes, Long Sleeper OR Short Sleeper Syndromes OR Sleeper Syndromes, Short OR Syndromes, Short Sleeper OR Short Sleep Phenotype OR Phenotype, Short Sleep OR Phenotypes, Short Sleep OR Short Sleep Phenotypes OR Sleep Phenotypes, Short OR Sleep-Related Neurogenic Tachypnea OR Neurogenic Tachypneas, Sleep-Related OR Sleep-Related Neurogenic Tachypneas OR Tachypneas, Sleep-Related Neurogenic OR Subwakefullness Syndromes OR Syndromes, Subwakefullness |
| --- | --- |
| #2 | Sensory Stimulation OR Multi-sensory Stimulation OR Music Therapy OR Therapy, Music OR Aromatherapy OR Aromatherapies OR Aroma Therapy OR Aroma Therapies OR Therapies, Aroma OR Therapy, Aroma OR Massage OR Zone Therapy OR Therapies, Zone OR Zone Therapies OR Therapy, Zone OR Massage Therapy OR Massage Therapies OR Therapies, Massage OR Therapy, Massage OR Phototherapy OR Phototherapies OR Light Therapy OR Light Therapies OR Therapies, Light OR Therapy, Light OR Photoradiation Therapy OR Photoradiation Therapies OR Therapies, Photoradiation OR Therapy, Photoradiation OR Blue Light Therapy OR Blue Light Therapies OR Light Therapies, Blue OR Light Therapy, Blue OR Therapies, Blue Light OR Therapy, Blue Light OR Blue-light Therapy OR Blue-light Therapies OR Therapies, Blue-light OR Therapy, Blue-light OR Red Light Phototherapy OR Light Phototherapies, Red OR Light Phototherapy, Red OR Phototherapies, Red Light OR Phototherapy, Red Light OR Red Light Phototherapies |
| #3 | #1 AND #2 |

Embase

| #1 | 'Sleep Disorders':ti OR 'Insomnia':ti OR 'Restless Legs Syndrome':ti OR 'Sleep Apnea, Obstructive':ti OR 'Disorders, Sleep Wake':ti OR 'Sleep Wake Disorder':ti OR 'Wake Disorders, Sleep':ti OR 'Disorders, Sleep':ti OR 'Sleep Disorder':ti OR 'Long Sleeper Syndromes':ti OR 'Sleeper Syndromes, Long':ti OR 'Syndromes, Long Sleeper':ti OR 'Short Sleeper Syndromes':ti OR 'Sleeper Syndromes, Short':ti OR 'Syndromes, Short Sleeper':ti OR 'Short Sleep Phenotype':ti OR 'Phenotype, Short Sleep':ti OR 'Phenotypes, Short Sleep':ti OR 'Short Sleep Phenotypes':ti OR 'Sleep Phenotypes, Short':ti OR 'Sleep-Related Neurogenic Tachypnea':ti OR 'Neurogenic Tachypneas, Sleep-Related':ti OR 'Sleep-Related Neurogenic Tachypneas':ti OR 'Tachypneas, Sleep-Related Neurogenic':ti OR 'Subwakefullness Syndromes':ti OR 'Syndromes, Subwakefullness':ti |
| --- | --- |
| #2 | 'Sensory Stimulation':ti OR 'Multi-sensory Stimulation':ti OR 'Music Therapy':ti OR 'Therapy, Music':ti OR 'Aromatherapy':ti OR 'Aromatherapies':ti OR 'Aroma Therapy':ti OR 'Aroma Therapies':ti OR 'Therapies, Aroma':ti OR 'Therapy, Aroma':ti OR 'Massage':ti OR 'Zone Therapy':ti OR 'Therapies, Zone':ti OR 'Zone Therapies':ti OR 'Therapy, Zone':ti OR 'Massage Therapy':ti OR 'Massage Therapies':ti OR 'Therapies, Massage':ti OR 'Therapy, Massage':ti OR 'Phototherapy':ti OR 'Phototherapies':ti OR 'Light Therapy':ti OR 'Light Therapies':ti OR 'Therapies, Light':ti OR 'Therapy, Light':ti OR 'Photoradiation Therapy':ti OR 'Photoradiation Therapies':ti OR 'Therapies, Photoradiation':ti OR 'Therapy, Photoradiation':ti OR 'Blue Light Therapy':ti OR 'Blue Light Therapies':ti OR 'Light Therapies, Blue':ti OR 'Light Therapy, Blue':ti OR 'Therapies, Blue Light':ti OR 'Therapy, Blue Light':ti OR 'Blue-light Therapy':ti OR 'Blue-light Therapies':ti OR 'Therapies, Blue-light':ti OR 'Therapy, Blue-light':ti OR 'Red Light Phototherapy':ti OR 'Light Phototherapies, Red':ti OR 'Light Phototherapy, Red':ti OR 'Phototherapies, Red Light':ti OR 'Phototherapy, Red Light':ti OR 'Red Light Phototherapies':ti |
| #3 | #1 AND #2 |

Web of science

| #1 | TI=(Sleep Disorders OR Insomnia OR Restless Legs Syndrome OR Sleep Apnea, Obstructive OR Disorders, Sleep Wake OR Sleep Wake Disorder OR Wake Disorders, Sleep OR Disorders, Sleep OR Sleep Disorder OR Long Sleeper Syndromes OR Sleeper Syndromes, Long OR Syndromes, Long Sleeper OR Short Sleeper Syndromes OR Sleeper Syndromes, Short OR Syndromes, Short Sleeper OR Short Sleep Phenotype OR Phenotype, Short Sleep OR Phenotypes, Short Sleep OR Short Sleep Phenotypes OR Sleep Phenotypes, Short OR Sleep-Related Neurogenic Tachypnea OR Neurogenic Tachypneas, Sleep-Related OR Sleep-Related Neurogenic Tachypneas OR Tachypneas, Sleep-Related Neurogenic OR Subwakefullness Syndromes OR Syndromes, Subwakefullness) |
| --- | --- |
| #2 | TI=(Sensory Stimulation OR Multi-sensory Stimulation OR Music Therapy OR Therapy, Music OR Aromatherapy OR Aromatherapies OR Aroma Therapy OR Aroma Therapies OR Therapies, Aroma OR Therapy, Aroma OR Massage OR Zone Therapy OR Therapies, Zone OR Zone Therapies OR Therapy, Zone OR Massage Therapy OR Massage Therapies OR Therapies, Massage OR Therapy, Massage OR Phototherapy OR Phototherapies OR Light Therapy OR Light Therapies OR Therapies, Light OR Therapy, Light OR Photoradiation Therapy OR Photoradiation Therapies OR Therapies, Photoradiation OR Therapy, Photoradiation OR Blue Light Therapy OR Blue Light Therapies OR Light Therapies, Blue OR Light Therapy, Blue OR Therapies, Blue Light OR Therapy, Blue Light OR Blue-light Therapy OR Blue-light Therapies OR Therapies, Blue-light OR Therapy, Blue-light OR Red Light Phototherapy OR Light Phototherapies, Red OR Light Phototherapy, Red OR Phototherapies, Red Light OR Phototherapy, Red Light OR Red Light Phototherapies) |
| #3 | #1 AND #2 |

Cochrane

| #1 | ((Sleep Disorders) OR (Insomnia) OR (Restless Legs Syndrome) OR (Sleep Apnea, Obstructive) OR (Disorders, Sleep Wake) OR (Sleep Wake Disorder) OR (Wake Disorders, Sleep) OR (Disorders, Sleep) OR (Sleep Disorder) OR (Long Sleeper Syndromes) OR (Sleeper Syndromes, Long) OR (Syndromes, Long Sleeper) OR (Short Sleeper Syndromes) OR (Sleeper Syndromes, Short) OR (Syndromes, Short Sleeper) OR (Short Sleep Phenotype) OR (Phenotype, Short Sleep) OR (Phenotypes, Short Sleep) OR (Short Sleep Phenotypes) OR (Sleep Phenotypes, Short) OR (Sleep-Related Neurogenic Tachypnea) OR (Neurogenic Tachypneas, Sleep-Related) OR (Sleep-Related Neurogenic Tachypneas) OR (Tachypneas, Sleep-Related Neurogenic) OR (Subwakefullness Syndromes) OR (Syndromes, Subwakefullness)):ti |
| --- | --- |
| #2 | ((Sensory Stimulation) OR (Multi-sensory Stimulation) OR (Music Therapy) OR (Therapy, Music) OR (Aromatherapy) OR (Aromatherapies) OR (Aroma Therapy) OR (Aroma Therapies) OR (Therapies, Aroma) OR (Therapy, Aroma) OR (Massage) OR (Zone Therapy) OR (Therapies, Zone) OR (Zone Therapies) OR (Therapy, Zone) OR (Massage Therapy) OR (Massage Therapies) OR (Therapies, Massage) OR (Therapy, Massage) OR (Phototherapy) OR (Phototherapies) OR (Light Therapy) OR (Light Therapies) OR (Therapies, Light) OR (Therapy, Light) OR (Photoradiation Therapy) OR (Photoradiation Therapies) OR (Therapies, Photoradiation) OR (Therapy, Photoradiation) OR (Blue Light Therapy) OR (Blue Light Therapies) OR (Light Therapies, Blue) OR (Light Therapy, Blue) OR (Therapies, Blue Light) OR (Therapy, Blue Light) OR (Blue-light Therapy) OR (Blue-light Therapies) OR (Therapies, Blue-light) OR (Therapy, Blue-light) OR (Red Light Phototherapy) OR (Light Phototherapies, Red) OR (Light Phototherapy, Red) OR (Phototherapies, Red Light) OR (Phototherapy, Red Light) OR (Red Light Phototherapies)):ti |
| #3 | #1 AND #2 |

CNKI

| Chinese | (SU='感官刺激' OR TI='感官刺激' OR KY='感官刺激' OR AB='感官刺激' OR SU='感觉刺激' OR TI='感觉刺激' OR KY='感觉刺激' OR AB='感觉刺激' OR SU='多感官' OR TI='多感官' OR KY='多感官' OR AB='多感官' OR SU='音乐疗法' OR TI='音乐疗法' OR KY='音乐疗法' OR AB='音乐疗法' OR SU='音乐干预' OR TI='音乐干预' OR KY='音乐干预' OR AB='音乐干预' OR SU='芳香疗法' OR TI='芳香疗法' OR KY='芳香疗法' OR AB='芳香疗法' OR SU='按摩' OR TI='按摩' OR KY='按摩' OR AB='按摩' OR SU='推拿' OR TI='推拿' OR KY='推拿' OR AB='推拿' OR SU='光疗法' OR TI='光疗法' OR KY='光疗法' OR AB='光疗法' OR SU='光照' OR TI='光照' OR KY='光照' OR AB='光照') AND ((SU='睡眠障碍' OR TI='睡眠障碍' OR KY='睡眠障碍' OR AB='睡眠障碍' OR SU='失眠' OR TI='失眠' OR KY='失眠' OR AB='失眠' OR SU='入睡困难' OR TI='入睡困难' OR KY='入睡困难' OR AB='入睡困难' OR SU='睡眠质量' OR TI='睡眠质量' OR KY='睡眠质量' OR AB='睡眠质量' OR SU='睡眠' OR TI='睡眠' OR KY='睡眠' OR AB='睡眠' OR SU='日间过度思睡' OR TI='日间过度思睡' OR KY='日间过度思睡' OR AB='日间过度思睡' OR SU='睡眠问题' OR TI='睡眠问题' OR KY='睡眠问题' OR AB='睡眠问题') OR (SU='睡眠呼吸暂停' OR TI='睡眠呼吸暂停' OR KY='睡眠呼吸暂停' OR AB='睡眠呼吸暂停' OR SU='不宁腿综合征' OR TI='不宁腿综合征' OR KY='不宁腿综合征' OR AB='不宁腿综合征' OR SU='快速眼动睡眠行为障碍' OR TI='快速眼动睡眠行为障碍' OR KY='快速眼动睡眠行为障碍' OR AB='快速眼动睡眠行为障碍')) |
| --- | --- |
| English | (SU='Sensory Stimulation' OR TI='Sensory Stimulation' OR KY='Sensory Stimulation' OR AB='Sensory Stimulation' OR SU='Sensory Activation' OR TI='Sensory Activation' OR KY='Sensory Activation' OR AB='Sensory Activation' OR SU='Multisensory' OR TI='Multisensory' OR KY='Multisensory' OR AB='Multisensory' OR SU='Music Therapy' OR TI='Music Therapy' OR KY='Music Therapy' OR AB='Music Therapy' OR SU='Music Intervention' OR TI='Music Intervention' OR KY='Music Intervention' OR AB='Music Intervention' OR SU='Aromatherapy' OR TI='Aromatherapy' OR KY='Aromatherapy' OR AB='Aromatherapy' OR SU='Massage' OR TI='Massage' OR KY='Massage' OR AB='Massage' OR SU='Tuina' OR TI='Tuina' OR KY='Tuina' OR AB='Tuina' OR SU='Light Therapy' OR TI='Light Therapy' OR KY='Light Therapy' OR AB='Light Therapy' OR SU='Light Exposure' OR TI='Light Exposure' OR KY='Light Exposure' OR AB='Light Exposure') AND ((SU='Sleep Disorders' OR TI='Sleep Disorders' OR KY='Sleep Disorders' OR AB='Sleep Disorders' OR SU='Insomnia' OR TI='Insomnia' OR KY='Insomnia' OR AB='Insomnia' OR SU='Difficulty Falling Asleep' OR TI='Difficulty Falling Asleep' OR KY='Difficulty Falling Asleep' OR AB='Difficulty Falling Asleep' OR SU='Sleep Quality' OR TI='Sleep Quality' OR KY='Sleep Quality' OR AB='Sleep Quality' OR SU='Sleep' OR TI='Sleep' OR KY='Sleep' OR AB='Sleep' OR SU='Excessive Daytime Sleepiness' OR TI='Excessive Daytime Sleepiness' OR KY='Excessive Daytime Sleepiness' OR AB='Excessive Daytime Sleepiness' OR SU='Sleep Problems' OR TI='Sleep Problems' OR KY='Sleep Problems' OR AB='Sleep Problems') OR (SU='Sleep Apnea' OR TI='Sleep Apnea' OR KY='Sleep Apnea' OR AB='Sleep Apnea' OR SU='Restless Legs Syndrome' OR TI='Restless Legs Syndrome' OR KY='Restless Legs Syndrome' OR AB='Restless Legs Syndrome' OR SU='Rapid Eye Movement Sleep Behavior Disorder' OR TI='Rapid Eye Movement Sleep Behavior Disorder' OR KY='Rapid Eye Movement Sleep Behavior Disorder' OR AB='Rapid Eye Movement Sleep Behavior Disorder')) |

Wanfang

| Chinese | 主题:("感官刺激" OR "感觉刺激" OR "多感官" OR "音乐疗法" OR "音乐干预" OR "芳香疗法" OR "按摩" OR "推拿" OR "光疗法" OR "光照") AND 主题:("睡眠障碍" OR "失眠" OR "入睡困难" OR "睡眠质量" OR "睡眠" OR "日间过度思睡" OR "睡眠问题" OR "睡眠呼吸暂停" OR "不宁腿综合征" OR "快速眼动睡眠行为障碍") |
| --- | --- |
| English | Subject: ("Sensory Stimulation" OR "Sensory Activation" OR "Multisensory" OR "Music Therapy" OR "Music Intervention" OR "Aromatherapy" OR "Massage" OR "Tuina" OR "Light Therapy" OR "Light Exposure") AND Subject: ("Sleep Disorders" OR "Insomnia" OR "Difficulty Falling Asleep" OR "Sleep Quality" OR "Sleep" OR "Excessive Daytime Sleepiness" OR "Sleep Problems" OR "Sleep Apnea" OR "Restless Legs Syndrome" OR "Rapid Eye Movement Sleep Behavior Disorder") |
