# Supplementary material for: The effects of sensory stimulation therapy in patients with sleep disorders: a scoping review
Source: Front Neurosci. 2025 Oct 3;19:1682267. doi: 10.3389/fnins.2025.1682267 (PMC12533477; doi:10.3389/fnins.2025.1682267)
Supplement: Supplementary file 2 [file Data_Sheet_2.docx]

**Supplementary Appendix B: the risk of bias assessment**

Given that all the studies included in this research are randomized controlled trials, the bias risk assessment tools from the Cochrane Systematic Review Manual were employed to evaluate the quality of the literature. A visual representation of the risk of bias assessment is shown in the following figures.


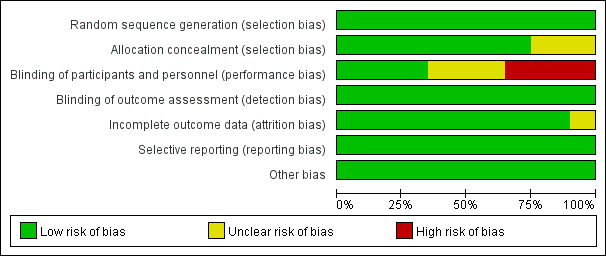


**Fig. 1.** Risk of bias of the included studies


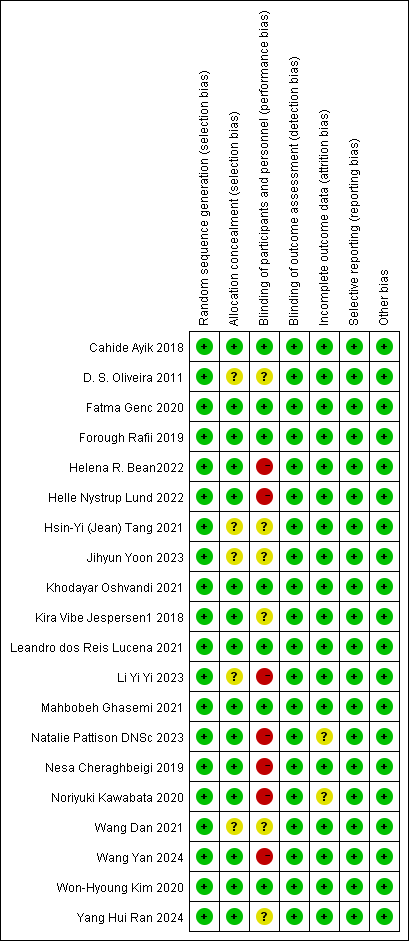


**Fig. 2.** Summary of Bias Risks in Included studies
